# The Biofabrication of Diseased Artery In Vitro Models

**DOI:** 10.3390/mi13020326

**Published:** 2022-02-19

**Authors:** Chen Pan, Qiqi Gao, Byoung-Soo Kim, Yafeng Han, Ge Gao

**Affiliations:** 1 Institute of Engineering Medicine, Beijing Institute of Technology, Beijing 100081, China; 3220195044@bit.edu.cn (C.P.); 3120211985@bit.edu.cn (Q.G.); 2 School of Mechanical Engineering, Beijing Institute of Technology, Beijing 100081, China; hanyafeng@bit.edu.cn; 3 School of Medical Technology, Beijing Institute of Technology, Beijing 100081, China; 4 School of Biomedical Convergence Engineering, Pusan National University, Yangsan 626841, Korea

**Keywords:** biofabrication techniques, arterial diseases, tissue microenvironment, in vitro models

## Abstract

As the leading causes of global death, cardiovascular diseases are generally initiated by artery-related disorders such as atherosclerosis, thrombosis, and aneurysm. Although clinical treatments have been developed to rescue patients suffering from artery-related disorders, the underlying pathologies of these arterial abnormalities are not fully understood. Biofabrication techniques pave the way to constructing diseased artery in vitro models using human vascular cells, biomaterials, and biomolecules, which are capable of recapitulating arterial pathophysiology with superior performance compared with conventional planar cell culture and experimental animal models. This review discusses the critical elements in the arterial microenvironment which are important considerations for recreating biomimetic human arteries with the desired disorders in vitro. Afterward, conventionally biofabricated platforms for the investigation of arterial diseases are summarized, along with their merits and shortcomings, followed by a comprehensive review of advanced biofabrication techniques and the progress of their applications in establishing diseased artery models.

## 1. Introduction

The vascular system in the human body plays an important role in circulating nutrients, oxygen, and metabolic wastes. In general, the vascular system can be divided into three subsets of blood vessels: arteries, veins, and capillaries [1]. Different types of blood vessels possess varied configurations of tissues for exerting specific functions. While the capillaries help to complete the exchange of supplies and metabolic products, the arteries and veins serve as highways to cyclically escort the bloodstream to each tissue and organ. When any member of the vascular system becomes disordered, a variety of fatal diseases and complications might be induced. For example, artery-relevant abnormalities (e.g., atherosclerosis, thrombosis, and aneurysm) can cause cardiovascular diseases, such as heart attack, stroke, and hemorrhage, which are the leading causes of death for millions of people worldwide each year [2,3].

Although an increasing number of newly developed clinical treatments have been demonstrated to be useful for rescuing patients (e.g., angioplasty, stent deployment, and bypass surgery), plenty of the underlying pathologies of these diseases remain unrevealed, limiting the establishment of effective therapies for disease regression and recovery [4]. In addition, the exploration of specific drugs is an essential way to alleviate or even reverse the diseases’ progression. However, to ensure clinical safety and pharmaceutic effect, drug discovery is usually a time-consuming and costly process, requiring approximately 10–15 years and 1.5 billion dollars for a new drug available in the market [5]. Therefore, the arterial disease model that can accurately reflect the pathophysiology of human arteries is urgently needed as a diagnostic and predictive platform to explain disease mechanisms and promote the exploration of specific drugs.

A plethora of diseased artery platforms have been previously developed based on experimental animals or planar cell culture [6]. However, both types of models face significant limitations that confine their applications. Regardless of the ethical concerns [7,8], diverse animal species, including rodents, mammals, and primates, have been utilized to create disease models related to human arteries. However, the interspecific differences between animals and humans inevitably result in pathological discrepancy and unpredictable variations of drug responses [9]. Hence, the outcomes acquired from experimental animals might provide limited or even invalid references for clinical applications. On the other hand, 2D cell culture mostly lacks the 3D microenvironments present in natural tissues, and thereby cannot correctly and precisely reflect the events occurring in the human body. For instance, the planar substrate not only inhibits the polarization and alignment of cells but also confines the interactions between multiple types of cells. In addition, the absence of key physiological dynamics (e.g., hemodynamics, muscle contraction, circulatory cells, and factors gradients) that highly influence cell fates leads to difficulties in controlling the disease initiation and progression [10]. To overcome these limitations, advanced in vitro platforms that can recapitulate arterial diseases are desperately anticipated.

To establish a viable diseased artery platform, it is essential to define the core features that should be involved. First, the model should recruit all relevant cells types associated with the target disease. Second, the involved multiple types of cells should be organized in a three-dimensional space following the anatomical architectures shown in natural tissues and disease nidus, facilitating the formation of functional tissues and cell–cell interactions. Third, important biomechanical and biochemical stimuli should be incorporated into the model, exposing cells to disease environments that are key factors in the initiation and deterioration of the relevant disorders. Last, the platform should be easy to reproduce via commonly used tissue culture and engineering approaches, and the outcomes should be easily obtained and analyzed. With the development and progress of science and technology, emerging biomanufacturing technologies may be able to fabricate in vitro models that can achieve these objectives. Biologists and engineers have collaborated to model arterial diseases in vitro, producing diverse innovative tools.

This review focuses on the biofabrication of diseased artery in vitro models. We first describe the critical elements in the vascular microenvironment, including vascular cells, cell–cell crosstalk, cell–ECM interaction, and physiological dynamics, which are important considerations for recreating biomimetic human arteries with desired disorders in vitro. Afterward, conventional platforms for the investigation of arterial diseases, such as designed apparatuses, transwell systems, and needle-templating bulk hydrogels are summarized along with their merits and shortcomings. Upon the discussions of advanced techniques (e.g., tissue-engineered blood vessels, organ-on-a-chip, and 3D bioprinting), the recent progress of diseased artery models (e.g., atherosclerosis, thrombosis, and aneurysm) established using these biofabrication approaches is reviewed.

## 2. Critical Elements in the Vascular Tissue Microenvironment

The tissue microenvironment refers to a dynamic population of multiple types of cells and non-cellular components (e.g., extracellular matrix (ECM), growth factors, and cytokines) which form a multi-faceted regulatory network that helps to maintain the homeostasis of tissues and organs [11]. Besides, in natural conditions, biomechanical (e.g., hemodynamics and muscle contraction) and biochemical stimuli (e.g., ion signaling and factor gradients) are ubiquitous in the human body, providing dynamic signals that guide cell behaviors and regulate tissue functions [12]. Therefore, it is necessary to understand the tissue microenvironment residing in the human artery before establishing relevant disease platforms.

### 2.1. Cells and ECMs in the Artery

**Figure 1:**
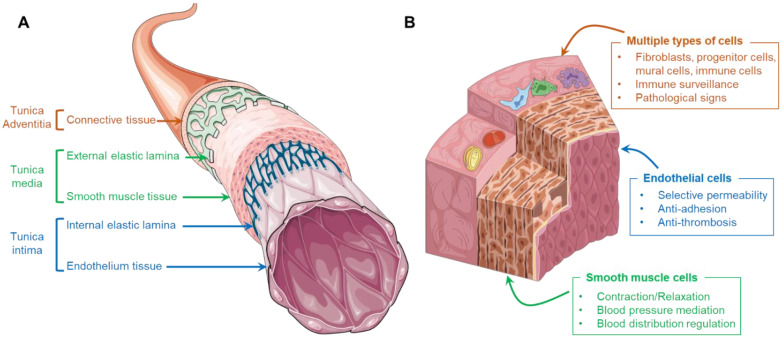
Schematic illustrations of arterial anatomy: (**A**) compartmentalized human arterial wall; (**B**) cell types in arterial tissues and relevant physiological functions. This figure was prepared using a template on the Sevier medical art website (https://smart.servier.com/, accessed on 12 July 2021).

In general, the wall of a human artery is composed of three distinctive layers, namely tunica intima, tunica media, and tunica adventitia, which are sequentially positioned from the luminal to the abluminal side (Figure 1A). The tunica intima consists of a monolayer of endothelial cells (ECs), also called the endothelium, that directly contact the bloodstream. These cells are anchored on an underlying basement membrane consisting of laminin, type IV collagen, nidogen, perlecan, and type XV and XVIII collagens [13]. Densely packed vascular smooth muscle cells (VSMCs), elastic tissue, and collagen fibers form the tunica media, wrapping around the tunica intima. As a shell of arteries, loose connective tissues containing collagen, osteopontin, and fibronectin synthesized by fibroblasts is the main component of tunica adventitia [14]. Although each layer of the artery involves different types of cells, the erratic migration of cells is strictly limited due to the presence of internal and external elastic membranes which are composed of abundant elastin and collagen. These membranes structurally and biologically separate the constituents, resulting in a clearly compartmentalized arterial wall.

The ECs residing in healthy arteries play an important role in mitigating blood coagulation and regulating molecular transportation. The typical physiological functions of the endothelium include selective permeability, the resistance of platelet and leukocyte adhesion, and stimuli response (Figure 1B). First, the endothelium consists of a monolayer of ECs that are closely connected by adhesion, tight, and gap junctions. The presence of these junctional proteins permits only the penetration of solutes, ions, and metabolites but prevents the passage of cells and macromolecules and is thus important in mediating vascular permeability and leukocyte extravasation. Moreover, the endothelium exhibits a quiescent phenotype that produces nitric oxide (NO) and generates an anti-thrombotic and non-adhesive surface that suppresses platelet aggregation, oxidative stress generation, and leukocyte adhesion. Furthermore, under different hemodynamic forces or biochemical signals, the ECs can exhibit corresponding responses (e.g., self-remolding, activation, and dysfunction). For instance, regular shear stress induced by laminar blood flow results in longitudinally aligned ECs at quiescent status, while a turbulent flow may induce the dysfunction of ECs that express cell adhesion molecules, facilitating interactions with circulating leukocytes [15].

The VSMCs are circumferentially arranged in the tunica media, playing an important role in regulating blood pressure and blood distribution by maintaining an intrinsic level of contractile force, known as vascular tone (Figure 1B) [16]. Unlike the muscle cells present in skeletal muscular tissue, the VSMCs are involuntary, non-striated cells. Despite the absence of innervation, the contraction of VSMCs can be triggered via two interlinked pathways, the calcium-dependent pathway and Rho/Rho-associated protein kinase pathway [17]. More importantly, under different conditions, VSMCs can perform both contractile and synthetic functions, which are characterized by changes in the morphology, proliferation, migration, and expression of different marker proteins (Figure 1B). In general, residing in mature and healthy arteries, VSMCs maintain a contractile phenotype (or differentiated phenotype), identified by elongated, spindle-like morphology that expresses contractile-specific markers, such as smooth muscle myosin heavy chain (SM-MHC), smooth muscle α-actin (SMA), and calponin [18]. These contractile cells are reluctant to proliferate and migrate, contributing to the homeostasis of arterial tissue. However, upon injury or pathological stimulation, the VSMCs de-differentiate and re-enter the cell cycle, exhibiting a synthetic phenotype that results in the active proliferation and massive synthesis of ECMs. The aberrant proliferation and migration of synthetic VSMCs might contribute to the development of arterial diseases (e.g., intima hyperplasia and the formation of fibrous atherosclerotic plaque).

Although the adventitia might not be the frontline during the initiation and progression of most arterial disorders, it acts as immune surveillance and warns of impending vascular diseases. Except for the dominated fibroblasts, the external layer of the artery also includes a mixture of progenitor/stem cells, pericytes, myofibroblasts, macrophages, and dendritic cells which respond to endothelial injury and travel into the other two inner layers for the missions of immune cell trafficking and ECM compositional correction (Figure 1B) [19]. In addition, it has been noted that the changes present in the adventitia (e.g., hypercellularity, promoted connective tissue production, and discernable inflammation) could be used to foresee relevant disorders. For instance, various forms of atherosclerosis, hypertension, and vascular injury are characterized by adventitial cell proliferation and increased leukocyte levels in the perivascular spaces [20].

### 2.2. Cell–Cell Crosstalk

Although the human artery is distinctively compartmentalized, the representative cells in each layer continuously communicate not only to maintain the vascular homeostasis but also to mediate diseases. The response of the artery toward pathophysiological stimuli highly depends on the crosstalk between ECs and VSMCs. Such intercellular communications are basically achieved by paracrine association via the diffusion/transportation of soluble factors and extracellular vesicles.

Soluble factors, including growth factors, small molecules, and cytokines, are commonly synthesized by cells with specific purposes. Since they are capable of binding to cellular receptors and causing permissive or preventive signaling events (e.g., cell proliferation, differentiation, apoptosis), the diffusion of soluble factors is an essential process in EC–VSMC crosstalk. In general, the factors secreted by ECs and VSMCs can regulate and control the morphology, phenotype, and function of each other. For instance, since VSMCs are not directly exposed to the biomechanical and biochemical changes in the blood flow, the signal transduction induced by ECs creates a bridge for VSMCs to react. One representative example is that the endothelial-derived factors can affect the contractile condition of VSMCs, leading to the mediation of vascular tone. While the nitric oxide, hyperpolarizing agents, and prostacyclin released from ECs can cause muscular relaxation in the artery, other factors such as angiotensin II or endothelin promote the contraction of VSMCs [21]. Except for the regulation of vascular tone, soluble molecules produced by ECs (e.g., heparan sulfate proteoglycans, platelet-derived growth factors, tissue growth factors B1, etc.) have demonstrated their capacities for modulating the phenotype switch, migration, and ECM deposition of VSMCs [22,23,24,25]. Reciprocally, the activity of VSMCs can influence the functions of ECs in the arterial microenvironment. Reported evidence has described that the VSMCs displaying a synthetic phenotype impose an anti-angiogenic effect on ECs, whereas the contractile VSMCs promote angiogenic activity through Notch signals [26]. A study also proved that the interaction of VSMCs with ECs helps to regulate the response of ECs toward flow signals and injuries via the vascular mammalian target of the rapamycin pathway [27].

Another important mechanism of intercellular dialogue lies in the secretion of extracellular vesicles. According to the dimension, lipid composition, or mechanisms of formation and discharge, the extracellular vesicles can be categorized into three types: exosome (30–100 nm), microvesicle (0.1–1 μm), and apoptotic bodies (over 1 μm) [28]. These extracellular vesicles can escort a cargo of proteins, microRNAs, lipids, and metabolites from the parent cells to the extracellular space, enabling a distinctive means of cell–cell interaction. In physiological circumstances, the healthy ECs release the extracellular microvesicles containing miR-143 and miR-145 for the maintenance of the contractile function of VSMCs, which contributes to regulating phenotypic target genes in these cells. As a response to vascular injury, the expression of miR-143 and miR-145 is drastically reduced, resulting in the phenotypic change of VSMCs to a synthetic lineage [29]. On the other hand, the miRNA-laden extracellular vesicles secreted by VSMCs were also found working as messengers to ECs under pathological conditions. For example, the presence of oxidized low-density lipoprotein (ox-LDL) in atherosclerotic progression can upregulate the exosome-mediated transfer of miR-155 in human aortic VSMCs. As such a change is received by ECs, the endothelial proliferation, migration, and re-endothelialization could be inhibited, leading to increased endothelial permeability [30].

Besides the interactions between parenchymal cells in arterial tissue, the crosstalk between arterial tissue cells and the cells circulating in the bloodstream or exiting in perivascular tissues also significantly determines the pathophysiology of the artery. The communication between ECs and leukocytes is a representative example. In normal cases, the ECs tightly contact each other via several types of intercellular junctions, forming a non-adhesive endothelial surface that limits the attachment and extravasation of patrolling white blood vessels. In response to inflammatory cytokines such as tumor necrosis factor α (TNF-α) and interleukin 6, the ECs are locally activated, expressing intercellular adhesion molecules (e.g., intercellular adhesion molecule 1 (ICAM-1), vascular cell adhesion molecule 1 (VCAM-1), P-selectin, E-selectin, etc.) [31]. These molecules present on the surface of ECs attract and enable the adhesion of circulating leukocytes on the endothelium, which thereafter transmigrate toward infectious sites. Closely related to endothelial activation, endothelial dysfunction, defined as the disability to produce NO, is usually triggered by the risk factors of arterial diseases (e.g., smoking, alcohol, hyperlipidemia, etc.) [32]. As the EC-derived NO can effectively prevent the recruitment of leukocytes by suppressing the expression of VCAM-1, ICAM1, and selectins, the endothelial dysfunction clearly promotes the interactions with leukocytes. These inflammation-responsive changes of ECs have been regarded as the initiation stage of various diseases, including atherosclerosis, coronary heart disease, hypertension, and hypercholesterolemia.

Therefore, due to the importance and complexity of intercellular communications, it is critical to involve all participating cells when establishing an in vitro model for investigating the pathological events and signaling pathways of artery-related diseases.

### 2.3. Cell–ECM Interaction

Toward the replication of vascular tissue microenvironments, the interaction between cells and ECMs is another pivotal consideration because the compositional and structural properties of ECMs significantly direct cell fates. Relying on transmembrane receptors, such as integrins, α-dystroglycan, and syndecans, such messages can be detected by cells, providing a link between the cytoskeletons and the ECMs to enable cell–ECM contact.

All parenchymal cells in the arterial tissue secrete matrix components that contribute to the maintenance of the vascular properties and affect adjacent cell functions. ECs are primarily responsible for the synthesis, deposition, and assembly of the basement membrane, which contains laminin, type IV collagen, perlecan, and nidogens. In return, the EC functions are correlated with the formation and degradation of the basement membrane. While the matured basement membrane is a prerequisite for ECs to generate stable endothelium tissues, the dynamic degradation of the basement membrane as a result of enzyme activity (e.g., matrix metalloproteinases) facilitates the migration of ECs, leading to the sprouting of micro-vessels [33]. VSMCs, in response to tissue injury, exhibit changes from a contractile to a synthetic phenotype, whereby the cells begin to secrete abundant ECM components, including collagen, elastin, proteoglycan, and glycoprotein to achieve tissue repair. However, the overgrowth of synthetic VSMCs might induce arterial wall dysfunction and vascular diseases.

Varied compositions of the matrix proteins are particularly associated with cellular receptors. Upon ligand binding, specific integrins determine different signal transduction pathways that mediate cellular fates, such as the modulation of the cell cycle, the organization of the intracellular cytoskeleton, and phenotypic switching. For instance, it is known that the phenotypic change of VSMCs is highly dependent on the type of ECM component. Among the primary ECM components present in tunica media, type I collagen and fibronectin induce a synthetic VSMC phenotype, while type IV collagen and assorted proteoglycans (e.g., heparin and perlecan) appear to inhibit the proliferation and migration of VSMCs [34]. Interestingly, the structures of ECMs also participate in the regulation of VSMC phenotypes. Although the monomeric collagen activates VSMC proliferation, the fibrillar type I collagen has been demonstrated to promote the contractile phenotype. In addition, the composition of collagen fibrils may affect the migration of VSMCs, which is related to different focal adhesion compositions and integrin functions [35].

### 2.4. Physiological Dynamics

As a part of the circulatory system for the transportation of pumped blood, the arteries are exposed to a variety of physiological dynamic signals including stresses and deformations originating from blood flow and pressure.

In general, the dynamic stress imparted on the arterial wall by blood flow is named hemodynamics, entailing the shear stresses from the flow of blood, longitudinal stresses from the surrounding tissue, and circumferential stress from the blood pressure. ECs possess diverse receptors that can sense the blood flow and pass hemodynamic signals through mechanotransductive signaling pathways to recipient molecules, leading to phenotypic and functional changes. In healthy arteries with simple straight geometries, the ECs are exposed to laminar flows and high shear stresses. Such a condition induces pro-survival antioxidant signals and maintains the quiescent phenotype of ECs. However, for bifurcated and curved arteries, the flows are usually disturbed, generating slow and oscillating shear stresses, where the ECs show a pro-inflammatory phenotype associated with low NO production, high permeability, pro-adhesiveness, pro-coagulation, and pro-proliferative properties [36,37]. For this reason, the sites with these architectures are susceptible to the initiation of arterial diseases, such as atherosclerosis and aneurysm. Although the VSMCs cannot directly detect the shear stress of blood flow, the endothelium helps to coordinate their response to this type of mechanical stimuli via cell–cell interactions, such as NO release.

In contrast to the shear stresses, stretch deformation directly influences VSMCs. Such a mechanical force is demonstrated to enhance the expression of ECMs and contractile proteins from VSMCs. Previous efforts have proven that the physiological mechanical strain applied to the VSMCs residing in the human adult aorta can increase the synthesis of collagen and fibronectin, as well as the levels of matrix-degrading enzymes, leading to the accumulation of ECMs. Enlightened by this finding, bioreactors that can provide mechanical stimuli have been integrated into the process of tissue engineering to produce artificial blood vessel grafts [38]. Besides the effects on medial tissue remodeling, upon the transduction of mechanical stress, the function of integrins is a key factor that affects the VSMC phenotype. For instance, the implementation of cyclic mechanical strain showed differential effects on the expression of SM-MHC by VSMCs from neonatal rats and adults, suggesting the inclined contractile phenotype [39]. Despite these findings, the response of VSMCs to mechanical stimuli remains conflicting, which could probably be attributed to the variations in the degree and features of the experienced stretches. Physiological stress promotes the contractile phenotype, whereas over-stressing or under-stressing provokes synthetic modulation. This controversy may reflect the intrinsic diversity of VSMCs as well as the plethora of responses toward different local microenvironments.

Taken together, similar to other tissues in the human body, the artery possesses an intricate microenvironment entailing multiple types of cells and ECM components. The close associations between cells and cells or cells and ECMs enable the achievement and modulation of arterial physiological functions. Besides, the physiological dynamics originating from blood flows clearly have a major impact on cellular fates and performance. Therefore, to establish an in vitro platform that is viable for recapitulating the pathophysiology of the human artery, all these critical elements should be collaboratively involved.

## 3. Conventional Diseased Vascular In Vitro Models

### 3.1. Designed Apparatus

Upon the maturation of the cell culture technique, it is possible to culture vascular cells in planar devices, such as culture dishes and flasks. However, the mono-type of cells cultured statically in two-dimensional space can hardly reflect the microenvironments in their natural counterparts. To overcome the challenges, referring to the working principle of the rheometer, the first generation of in vitro apparatus was designed to understand the response of vascular cells to tunable flow shear stress.

The designed apparatus is made up of cones and plates to simulate the viscoelasticity, the fluid shear stress of ECs in vitro, and other properties of blood [40]. For instance, a cone-and-plate device and a two-disc rheometer and orbital shaker for the induction of flow in multi-well cell culture dishes. The cone shaft is connected via a flexible coupling to a motor with a feedback controller that ensures accurate speed control over long periods. The device appears when the cover plate is in position and secured via the spring-loaded clamps, allowing the use of stable, time-varying, and directional laminar flow, or dynamic shear stress.

**Figure 2:**
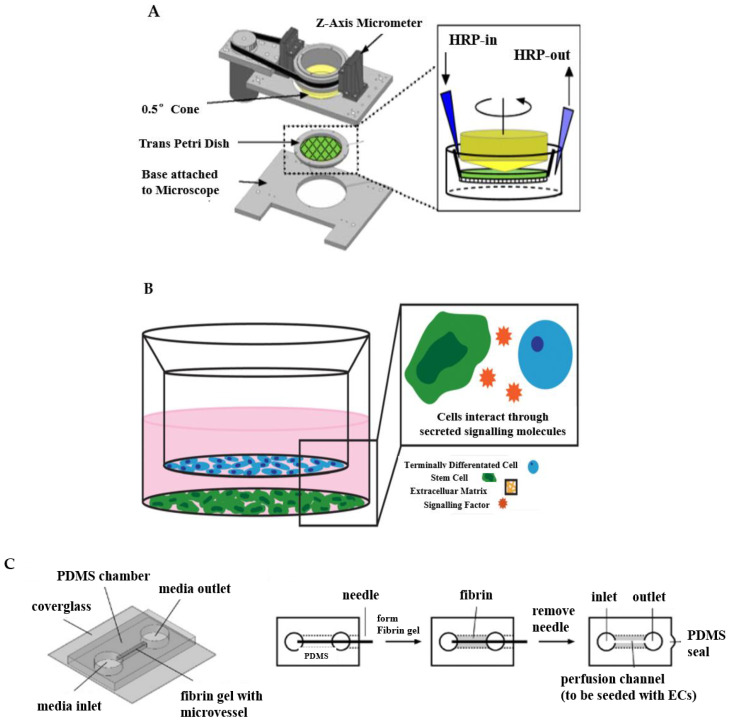
Conventional manufacturing methods of diseased vascular in vitro models: (**A**) designed apparatus. Reprinted with permission from ref. [43]. Copyright 2022 Copyright Orr et al.; (**B**) transwell. Reprinted with permission from ref. [44]. Copyright 2022 Copyright Nikolaos et al.; (**C**) needle-templating microchannels. Reprinted with permission from ref. [45]. Copyright 2022 Copyright Wong et al.

Such a type of design helps to reveal the relationships between EC activity and hemodynamic forces. Dewey et al. developed a cone-plate Couette flow device that was similar to commercial cone-plate viscometers consisting of the rotating cone and the stationary flat plate that first reported the response of ECs to flow shear stress [41]. They found that aligned endothelial monolayers tended to become reoriented by exposure to 8 dyne/cm^2^ shear for 72 h when rotating the flow direction 90° in the cone-plate device. By further experiments, the shape of cells was induced by the shear effect. These results also indicated that cell morphological change and orientation are quite sensitive to the applied shear force. Malek and teammates developed a cone-plate viscometer apparatus which was specially designed for studying the effect of fluid shear stress on large populations of adherent cells in vitro [42]. They cultured the bovine aortic endothelial (BAE) cells in Dulbecco’s modification of Eagle’s medium (DMEM) supplemented with 10% calf serum, and the BAE cells were exposed to 15 dyn/cm^2^ for 24 h and 96 h. The experimental results showed that compared with cells under static conditions, there was no difference between BAE cells exposed to 24 h of shear, as shown by the continual integrity of the BAE monolayer and frank alignment of cells along a streamlined direction when BAE cells were exposed to 96 h of constant fluid shear in the steady laminar regimen. With the assistance of the devised apparatus, the investigations of mechanisms in arterial diseases can be promoted to a higher level. Wayne Orr et al. reported that fluid flow, atherogenic flow, oxidized low-density lipo-protein (LDL), and proatherogenic cytokines all stimulated p21-activated kinase (PAK) phosphorylation and recruitment to cell–cell junctions based on large transwell membranes mounted into a modified cone-plate viscometer, as shown in Figure 2A [43]. They demonstrated that matrix-specific PAK activation mediated increased vascular permeability in the development of atherosclerosis.

Although the method mimics blood dynamics, there are huge limitations when building in vitro models of blood vessels simulated by a cone-plate apparatus. Because monolayer cells undergo the largest shear stress gradient from zero in the center to the periphery, this change in shear stress makes cone-plate and double-disc rheometers unsuitable for most biological studies. Cone-plate devices are usually used for monolayer cell studies, but rarely for the co-culture of cells. Therefore, 3D tissue culture is not achievable with this type of device, which limits the applications of this type of device.

### 3.2. Transwell System

To meet the requirement of co-culturing multiple types of cells in a single platform, the transwell system emerges as a useful tool, as shown in Figure 2B [44]. The transwell system consists of multi-well plates and porous membranes [46,47]. The porous membrane separates the well into an upper and a lower chamber [48]. Different cell culture media are placed in the upper and lower chambers to individually cultivate appropriate cells, which can be seeded either on the surfaces of the membrane or the substrate of the wells. Thus, the transwell system can enable the co-culture of two or three different cell types, thereby paving a way to emulating the tissue interfaces or complex microenvironments [47,49].

Benefiting from these advantages, the transwell system has been widely carried out to build diseased vascular in vitro models including atherosclerosis [49], the blood−brain barrier (BBB) [50], and stem cell-based co-culture [51,52]. Endothelial barrier functions have been recapitulated and evaluated using transwell systems. For instance, Stone et al. used 12 well plates to simulate the BBB in vitro model, and cultured astrocytes, pericytes, brain microvascular endothelial cells (HBMECs), and neurons in the orifice plates [53]. Their findings suggested that connections between endothelial cells, astrocytes, and pericytes improved using a larger membrane aperture. In their research, astrocytes were seeded on the basolateral side of the inserts, and pericytes on the bottom of the culture plate. Finally, astrocytes and pericytes were seeded on the outer base of the inlay in a mixed culture. Bicker et al. reported that a more rigorous barrier with better endothelial barrier function obtained using the transwell system could be co-cultured with astrocytes and/or pericytes in single, co-cultured, or triple cultures in contact and non-contact formats [54]. In addition, the triple-compartment cell co-culture model can be further customized and enhanced by introducing more complex or alternative immune/inflammatory components. Beyond the emulation of the arterial tissue divisions, an advanced transwell system is also capable of considering physiological dynamics. In a representative example, the basal membrane of the transwell insert was conferred with elasticity, which could be deformed to provide stretching strain to VSMCs [54,55,56]. The stretchable transwell device had three polydimethylsiloxane (PDMS) layers consisting of a hole-punched top layer, a thin middle membrane, and a patterned bottom layer. Before stretching, the chambers and channels were filled with water and the well bottom kept flat. By blocking one side of outlets while connecting the opposite side of outlets to the syringe pump, the flat surface of the substrate became a spherical cap that can cause the deformation of the membrane after injecting a certain volume of water, which provided stretching strain to VSMCs on the membrane.

The transwell system has been widely used in academia and the pharmaceutical industry because of its simplicity, cost-effectiveness, high and medium-throughput screening characteristics, and versatility. The transwell model not only has access to apical and basolateral (basal) compartments for drug applications and media sampling but also enables the visualization of cells during experiments. Despite the development of new biofabrication technologies, the transwell system still offers a distinct advantage in that it is relatively easy to set up and control and provides a range of endpoint studies. It provides a reliable, non-invasive quantitative measure of barrier integrity that can be repeated over the required time cycle with minimal damage to cells. However, a considerable drawback of the transwell system is the limited ability to offer flow signals to the residing cells. Therefore, it might be difficult to reveal the effects of hemodynamics on the pathological changes of arterial cells.

### 3.3. Needle-Templating Microchannels

The concept of creating microchannels to replicate vascular microenvironments truly realizes the in vitro models of arterial tissues and diseases on a three-dimensional level. To achieve this objective, needle-templating is a simple and maneuverable approach, which is also called the subtractive method [57]. This method generally inserts a micro-needle into a geometric model of a biomaterial such as a gel. After the material is stabilized, the needle is extracted from the bio-substrate, leaving behind a microtube that can be used to simulate an in vitro model of blood vessels, as shown in Figure 2C [45]. Initially, only a single microchannel was fabricated via combining the needle-templating [58,59]. By changing the arrangement of needles, the method of needle-templating also fabricated relatively complex multi-microchannels by arranging microneedles in arrays (either side-by-side or atop each other) [57,60]. Many studies have demonstrated the practical application of needle-templating microchannels in preparing in vitro models of arterial vessels [61,62]. Rigid needles of different diameters produce linear cylindrical channels, while flexible polydimethylsiloxane (PDMS) rods allow the manufacture of branched channels that can be endothelialized, aggregated, and perfused.

This approach has been widely used to study diverse pathophysiology in vitro models. Paek and colleagues focused on the preparation of infusible hydrogel cell culture scaffolds using needle-templating [63]. During the fabrication of the scaffolds, because two parallel microchannels physically guided the needles to slide along the entire length of the device, there was no significant deflection when the needle was inserted into the access port. In the next step, the fibrinogen solution moved along the cell culture chamber without moving the inserted needle and filled the entire cell culture chamber within a minute. Based on these works, they developed stem cell-derived physiological models of vascularized human adipose tissue and the blood-retinal barrier; moreover, they leveraged this to construct a 3D organotypic model of vascularized human lung adenocarcinoma. They successfully simulated the intravascular delivery, tumor-killing effects, and vascular toxicity of a clinical chemotherapeutic agent. Park et al. inserted the microneedle with an inner diameter of 0.38 mm and outer diameter 0.6 mm into the middle of the PDMS side walls as a microcapillary to fabricate microporous cell-laden agarose gels containing a microchannel [64]. They conducted in vitro experiments to verify the viability of human liver cancer cells grown in microporous agarose gel. Buchanan et al. developed a three-dimensional in vitro microfluidic vascular model to culture the tumor and endothelial cells under varying flow shear stress conditions [65]. They poured the neutralized collagen solution into a fluorinated ethylene propylene (FEP) tube, which was concentrically fitted with a 22 G stainless steel needle and sealed with a PDMS sleeve to prepare 3D microfluidics collagen hydrogel. The collagen was polymerized at 37 °C for 20 mins and then the needle was removed to form cylindrical microchannels embedded in the hydrogel. In the research field of the mechanism of how physiological dynamics influence endothelial cell function, Chen’s team introduced steel acupuncture needles of 0.16 mm into microfluidic devices to fabricate perfused microvessels [66]. After UV sterilizing for 20 min, the concentration of 2.5 mg/mL of collagen solution that was buffered with 10× DMEM and titrated to pH 8.0 with NaOH was injected into devices and polymerized for 20 min at 37 °C. They identified that Notch1 transmembrane receptor activation directly regulated vascular barrier function through a noncanonical, transcription-independent signaling mechanism. The shear stress triggered dLL4-dependent Notch1 proteolytic activation, revealing the Notch1 transmembrane domain, a key domain that mediated barrier establishment. Their work provides a mechanism for regulating vascular barrier function, establishing a previously unrecognized atypical cortical signaling pathway for Notch1.

However, there are many disadvantages and limitations of needle-templating microchannels. For instance, removing rigid needles and flexible rods may result in micrometer-scale defects at the interface, requiring extra care and precision in the removal process [67]. The insertion and removal of the needles have to be done manually, which adds an element of human error beyond human control, causing the vascular model to collapse. Furthermore, it is difficult to fabricate vascular models with cross-channel structures using needle-templating, and the mechanical properties of the cross structure prepared are poor. When large amounts of fluid are injected into the cross microchannels, high transient pressures are created that disrupt the hydrogel matrix and/or vascular system, and the vascular models often leak under high pressure and long-term perfusion. Especially, this method can hardly produce a bionic structure, and it is difficult to simulate the real bionic environment of a vascular model in vitro.

## 4. Emerging Biofabrication Techniques

Over the past decade, the improvement of micromanufacturing technology has promoted the development of tissue and organ models in vitro to overcome the shortcomings of traditional methods. The innovative bio-manufacturing technology has further contributed to the understanding of the pathology and clinical drug development of human diseases through fabricating sophisticated in vitro models of diseased arteries. The novel technologies have become a popular tool for revealing the pathologies at cellular and molecular levels, especially in the vascular system, whose characteristic mechanics and structures are difficult to generalize in traditional systems. This section reviews advanced biofabrication approaches including tissue-engineered blood vessels, organ-on-a-chip, and 3D bioprinted tissue analogs, focusing on the work principles of these manufacturing methods and discussing the benefits and limitations of these methods in building vascular pathological models in vitro.

### 4.1. Tissue-Engineered Blood Vessels

**Figure 3:**
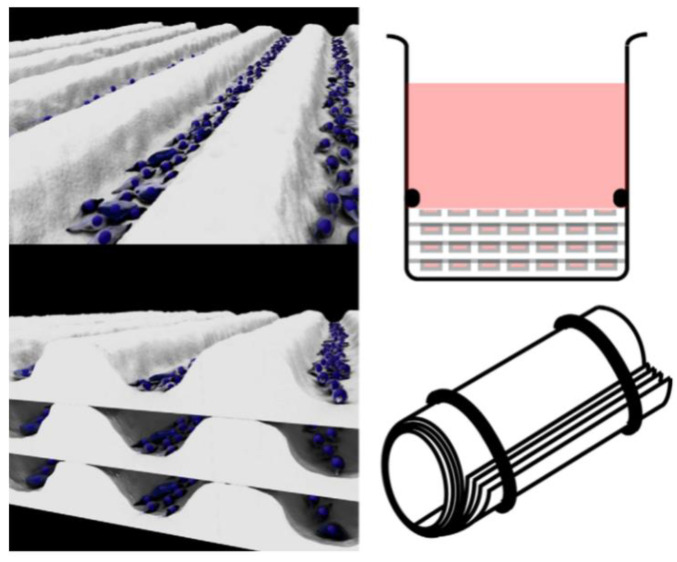
The TEBV method to fabricate a multilayer tube. Reprinted with permission from ref. [68]. Copyright 2022 Copyright Papenburg et al.

Translating the tissue-engineered blood vessels (TEBVs) that are commonly used as bypass grafts to understand the pathology of artery-relevant diseases is another strategy. The most representative approach for the development of TEBVs relies on the rolling of multiple vascular cell sheets to mimic the natural arrangement of cells and the structure and function of a variety of tubular tissues occurring naturally in the body [68,69] (Figure 3).

Due to mimicking and controlling the different cell arrangements [70], TEBV technology has the capacity to fabricate arterial-like structures in vitro using endothelial cells, smooth muscle cells, and fibroblasts [71]. Yuan et al. used a stress-induced rolling membrane technology to fabricate tubular structures with multilayered walls made of multiple types of orientated cells [72]. They used cured PDMS as the top membrane and stretched it through a mechanical stretcher to generate stress. The bottom membrane was relaxed, semi-cured PDMS. Different types of cells were seeded into the microfluidic channels and the cell sheet was rolled up to form a tube. The ECs were encased in circumferentially orientated SMCs to form the tunica media. Longitudinally orientated SMCs formed the tunica media and randomly orientated fibroblast cells formed the tunica adventitia. This method can be widely used to produce tubular tissues due to its high structural similarity and stable shape. In addition, the strategy enriches the toolbox of 3D micro/nanomanufacturing, moving from the initial 2D to 3D. Rolle et al. developed spatially controlled TEBVs by fusing SMC ring units into vascular structures with heterogeneous components similar to those observed in intimal hyperplasia or atherosclerosis [73]. Cho et al. fabricated atherosclerotic three-layer vessels with adjustable geometry to form a monolayer of fused endothelial cells and a condensed SMC layer using a cellular printing technique [74].

Undoubtedly, TEBVs might be the most eligible candidate for emulating the natural arterial function and physiological environment. However, the low reproducibility and production rate are the critical drawbacks of TEBVs as in vitro research models, especially when the applications require high throughput performances, such as drug screening.

### 4.2. Organ-on-a-Chip

**Figure 4:**
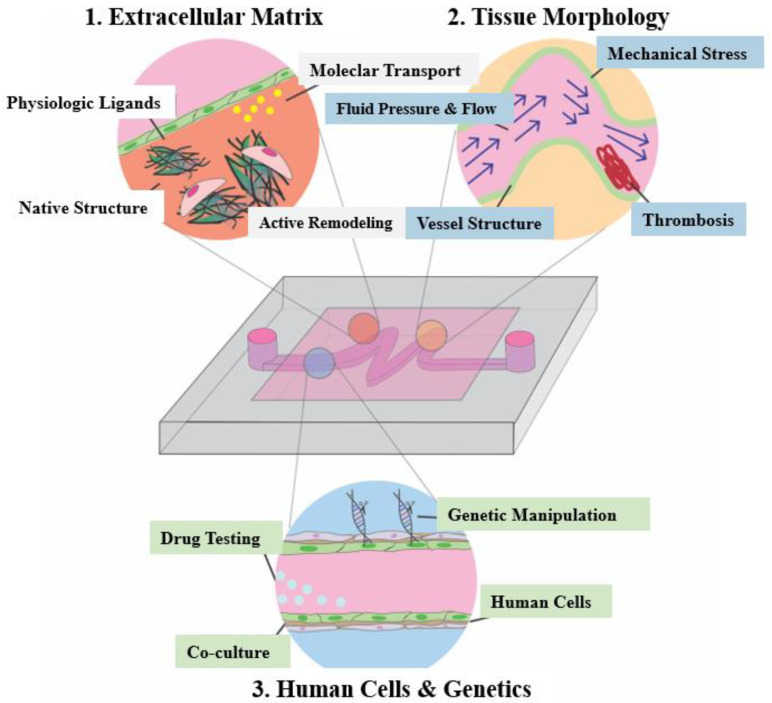
OoC device in vitro model of cardiovascular disease. Reprinted with permission from ref. [9]. Copyright 2022 Copyright Doherty et al.

An organ-on-a-chip (OoC) is a microfluidic cell culture device containing a continuous perfusion chamber with a multi-cellular layer structure, tissue interface, physicochemical microenvironment, and human vascular circulation. It can also be considered as a cell culture biomimetic system that can simulate and reconstruct the physiological functions of human organs, with the ability to adjust key parameters such as concentration gradients, shear forces, cell patterns, tissue boundaries, and tissue-organ interactions. OoC consists of four key components [75], including: (1) tunable microfluidic signals that mimic the physiological stimulations of blood flow and body fluid, (2) organized living cells that simplify the anatomy of the target tissue and organ, (3) convenient administration windows for providing different signal stimuli, and (4) sensing components used to detect and compile data, which can be embedded sensing output components or visual function evaluation systems based on transparent chips [76]. Figure 4 shows the simple application of OoC devices in the pathophysiological modeling of cardiovascular diseases [9].

OoC has exhibited distinguished advantages over the traditional 2D cell culture methods, such as realizing 3D cell co-culture, offering dynamic stimulations, and replicating the tissue interface. The presence and integration of multiple cell types could enable the elaborated chip to reflect the natural physiological interplays of cells (such as parenchymal cells, stromal cells, vascular cells, and immune cells) in a 3D environment. Besides, the OoC may also reconstitute the biomechanical characteristics of arterial tissue, such as the muscular contraction and the shear force induced by blood flow. In addition, the microchannels established on OoC serve to refresh and transport cell metabolite products [77]. Moreover, when biomaterials (e.g., bulk hydrogel and porous scaffolding) are incorporated with the OoC to emulate the tissue matrix, the 3D spatial arrangement of tissue interfaces can be replicated to study the response of cells to irregular ECM geometry under external mechanical stimulation.

Benefiting from these merits, OoC has shown broad potential for the construction of in vitro arterial models as well as applications in pathological investigation and drug screening [78]. Tsai et al. et al. fabricated an “endothelialized” vascular OoC, which reproduced and integrated a set of pathophysiological processes such as thrombosis [79]. Under controlled blood flow conditions, the model could quantitatively study how biophysical changes in blood diseases jointly led to microvascular occlusion and thrombosis. The microfluidic platform can also simulate and reproduce the altered tissue morphology and abnormal hemodynamics related to cardiovascular diseases. Neelamegham and teammates also investigated and examined the effects of platelet adhesion and cytoskeletal tension on thrombosis dynamics using a microfluidic-integrated microclot array elastometry (clotMAT) system by integrating a collagen microtissue array-based mechanical sensing platform with platelet flow [80]. The system reproduced the dynamic process of platelet adhesion and thrombus formation under different flow conditions and reported the changes of mechanical properties of the thrombus in real-time. Westein et al. built a microfluidic device to recreate the vascular biological environment of stenosis and used it to study how factors such as mural stress, stenosis geometry, and clotting factor activity change along with the stenosis structure [81]. Complementary in vitro studies using microfluidic narrow lumen showed enhanced platelet aggregation in the transparent outlet region with 60–80% channel obstruction within the shear rate range of the input tube wall. Günther et al. developed an OoC to focus on facilitating the evaluation of resistance artery structure and function under physiological conditions [82]. Compared to conventional pressure myography systems, the established OoC allowed for the loading, precise placement, and fixation as well as the controlled perfusion and superfusion of a small artery segment. For the OoC aiming at mimicking the diseased artery, Zhang and Neelamegham also comprehensively summarized the state of the art and their applications in the field of thrombosis and hemostasis [83].

Besides the shinning points discussed above, OoC may also pave the way for tackling the challenges of bioengineering techniques, such as guiding cellular compartmentalization and organization, as well as integrating bioelectrical and biochemical signals to provide the cells with a habitat comparable to their natural counterparts. For example, smooth muscle cells’ (SMCs) circumferential arrangement in the artery can be realized by preparing rectangular microchannels to investigate the relationship between cellular orientation and their functional contractility [84]. The narrow microchannels or grooves in some cases inhibit the cell proliferation necessary for tissue formation [85]. Choi et al. used OoC technology to prepare circular microchannels with orthogonal micro-grooves perpendicular to the axis of microchannels [86]. Such orthogonal microfolds embedded in the curvature surface of circular microchannels can lead to the circumferential arrangement of SMCs during culture.

To gain insight into the type and size of the intracellular forces generated by cell–cell and cell–ECM interactions, OoC can stimulate arterial deformation via electrodes, which has a significant impact on cell function in vitro [87]. The in vivo situation is transformed into an in vitro system by manipulating the stiffness of the ECM matrix with microelectrodes to reproduce cellular and subcellular forces. The contractility of each cell line can be individually controlled by local electrical stimulation with a microelectrode array [87]. The integrated electrodes with a microfluidic platform for measuring cross-epithelial resistance (TEER) has been widely used to evaluate EC integrity and barrier function [88].

Despite the great potential and achievements, there are limitations of current OoC systems that confine the extent of pathophysiological remodeling in vascular disease. A large number of developed OoC systems are based on the use of polydimethylsiloxane (PDMS). However, a major drawback of PDMS is that the material absorbs small hydrophobic molecules, making it difficult to evaluate the pharmacokinetics of drugs and toxins [89,90]. Although biocompatible hydrogels have been alternatively applied to build OoC, the long-term structural instability caused by their rapid degradation rate and swelling ratio drastically limits their performances. In addition, OoC models may not always include the same array of cells as in the body. They are often designed as rectangular channels stacked on top of each other or side by side, which makes them impossible to reproduce the exact flow within the cylindrical vessels. This may also alter the endothelial function and affect cell contraction-related mechanisms [91]. Another major obstacle is that the cells used in these model systems may not always represent the phenotype of human disease or the patient’s local environment, so the standardization of cell lines and growth protocols is necessary [89].

### 4.3. Three-Dimensional Bioprinted Organotypic Constructs

**Figure 5:**
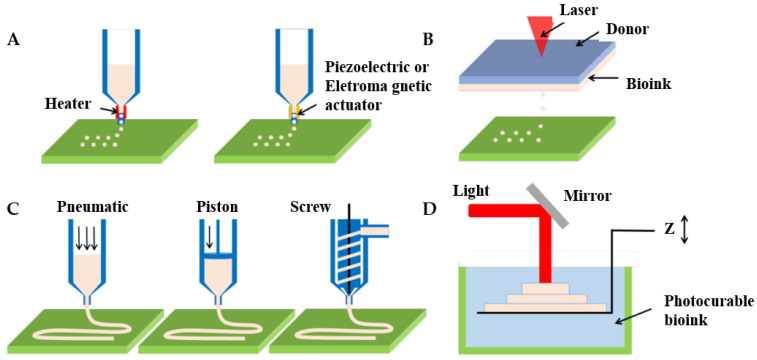
Schematic of bioprinting methods. (**A**) Inkjet bioprinting; (**B**) laser-assisted bioprinting; (**C**) extrusion bioprinting; (**D**) photopolymerization-based bioprinting. Reprinted with permission from ref. [93]. Copyright 2022 Copyright Gao et al.

Three-dimensional bioprinting represents the frontier technology in the field of biological manufacturing and is used to fabricate diseased vascular models with complex structures. A notable feature of 3D bioprinting is the ability to manufacture a wide variety of tubular structures and sizes, from arterial to arteriolar sizes, as well as branching structures, and even incorporating vascular preprocessing networks in 3D stents for tissue regeneration [91]. Dellaquila et al. [78] focused on reviewing the application of bioprinting technology in the in vitro models of blood vessels. They divided 3D bioprinting technology into bioprinting and bioassembly according to different manufacturing units. Current 3D bioprinting technologies are classified into inkjet bioprinting, laser-assisted bioprinting, extrusion-based bioprinting, and photopolymerization-based bioprinting [78,92], as shown in Figure 5 [93].

#### 4.3.1. Inkjet Bioprinting

Inkjet bioprinting (IBP)—consisting of X, Y, and Z, three moving modules with nozzles on the Z-axis for loading bioinks—uses heat or pressure electricity to deposit the bioink drop by drop, so it comes in two forms, including thermal inkjet bioprinting and piezoelectric inkjet bioprinting [92,94]. The thermal print head locally heats the bioink, creating a bubble that pushes water droplets through the nozzle. For piezoelectric inkjet bioprinting, vibration is the origin of droplet deposition in the case of piezoelectric and acoustic actuators [78]. IBP is a low-cost, fast printing technology with a high resolution of up to 50 μm. This technology is suitable for printing low viscosity biomaterials with a viscosity range of 3.5 to 12 mPa/s and low cell density (<10^6^ cells/mL). The applied bioinks can be chemically gelled or photo-crosslinked. Besides, its preparation time is low and print speed is fast, up to 10,000 droplets per second. Cell viability has been reported in the 80–95% range using this method due to temperature and mechanical stress [95].

IBP has been used in a variety of applications, including tissue engineering and regenerative medicine, transplantation and clinical applications, drug testing and high-throughput screening, and cancer research [94,96]. Because the vascularization of engineered tissue construction has been elusive and remains a barrier to the creation of functional replacement human organs, Dai et al. engineered capillaries using IBP by angiogenesis and the sprouting of endothelial cells between two parallel collagen-constructed vascular flow channels [97,98]. The angiogenic sprouting of vascular networks was absolutely important for clinically relevant tissue construction.

The advantages of thermal inkjet printers are fast printing speed, low cost, and wide availability. However, the risk of cells and materials being exposed to thermal and mechanical stresses, uneven droplet sizes, frequent clogging of nozzles, and unreliable cell encapsulation bring considerable disadvantages to the use of these printers in 3D bioprinting. The advantages of piezoelectric inkjet printers include the ability to generate and control uniform droplet sizes and jet directions, as well as the ability to protect cells from exposure to heat and pressure stress sources by creating a sound wave inside the print head that breaks liquid into droplets at regular intervals. In addition, the use of an open pool nozzle-free injection system can avoid the pure stress imposed on the wall cells at the tip of the nozzle. This reduces the potential loss of cell vitality and function and avoids the problem of nozzle clogging. Sonic injectors can be combined into multiple injectors in an adjustable array format, making it easy to print multiple units and material types simultaneously [99,100]. However, IBP also has material viscosity limitations because of the excessive force required to spray droplets using highly viscous solutions [101]. Another common drawback of IBP is that biomaterials must be in liquid form to form droplets. Therefore, the printed liquid must form a solid 3D structure with structural organization and function [92].

#### 4.3.2. Laser-Assisted Bioprinting

Laser-assisted bioprinting (LAB) is an on-demand drop method based on a pulsed laser beam incident on a donor slide in contact with the energy absorption layer. When a bioink is placed next to the energy-absorbing layer, a shock wave forms a jet of bioink that is deposited as water droplets on the collector’s slide [102,103]. Typical LAB consists of a pulsed laser beam, focusing systems in which a “ribbon” donation of transport support is usually made of glass covered with a layer of laser-energy-absorbing material (e.g., gold, titanium) and a layer of biological material (e.g., cells and/or hydrogels) ready in a liquid solution, as well as a ribbon-facing receiving substrate [92,94]. LAB results in laser-induced forward transfer effects, printing different living cells and biomaterials with precise and micron resolution [104]. LAB is a high-cost technology with a printing speed of 200–1600 mm/s, and deposits cells at a density of up to 10^8^ cells/ml with microscale resolution of a single cell per drop using a laser pulse repetition rate of 5 kHz [103]. Additionally, LAB is compatible with biomaterials with a viscosity of 1–300 mPa/s and is capable of printing mammalian cells with negligible effects on cell viability and function [105,106].

Given the characteristics of LAB manufacturing technology, Guillotin et al. used LAB to print stable, soft, free-form structures with a resolution compatible with the size of microvessels [103]. They placed a fibrinogen solution (90 mg/mL) as tissue paper on a glass substrate. Bioink made from thrombin and CaCl_2_ solution was packed into the ribbon. The bioink was then printed onto the fibrinogen, and the laser scanning speed was set to 200 mm/s, based on parallel and vertical lines, and the pattern was printed in seconds. Koch et al. used LAB technology to print NIH 3T3 and HaCaT cells in a collagen matrix to demonstrate tissue formation under natural conditions [105,107]. Different histological and immunohistological methods have shown that cells can proliferate throughout the entire region of the structure while maintaining their vigor and printing pattern.

Key advantages of LAB are high resolution, a wide range of material densities, and the ability to print batteries on solid or liquid substrates. Other benefits include automation, reproducibility, and high throughput. However, this is a very expensive technique and can cause cell damage. The high resolution of LAB requires fast gel dynamics to achieve high shape fidelity, which results in relatively low overall flow rates [108].

#### 4.3.3. Extrusion-Based Bioprinting

Extrusion-based bioprinting (EBB), as the most common 3D bioprinting method, uses layer stacking to produce in vitro models by depositing polymers or bioinks (such as tissue spheroids, cell pellets, microcarriers, decellularized ECM (dECM) components, and cell-laden hydrogels) [109,110]. An EBB system typically consists of one or more movements that can move along the X, Y, and Z axes, nozzles with different sizes [111], temperature-controlled material handling, dispensing systems, and receiving platforms. Furthermore, some bioprinters are equipped with a fiber optic light source to illuminate the deposition area and/or for photo-initiator activation. EBB is compatible with a wide range of biomaterials, including materials such as hydrogels, biocompatible copolymers, and cell spheres [112]. EBB with multiple nozzles enables the simultaneous printing of multiple materials. Importantly, for non-Newtonian materials, due to the properties of this material, the viscosity decreases in response to an increase in shear rate [113]. The high shear rate at the nozzle allows these materials to flow through the nozzle during the biomanufacturing process, and as they deposit, the shear rate decreases, resulting in a dramatic increase in viscosity. Extrusion biotechnology also has the capability for flexible printing structures by inputting G codes or CAD graphics slices. The microextrusion system for high resolution allows bioprinters to precisely manufacture complex structures designed using CAD software and facilitate patterns of multiple cell types. Besides, EB provides continuous material deposition and has a wider viscosity range of printed materials, typically from 30 mPa/s to more than 6 × 10^7^ mPa/s [92].

EBB has broad utility in various application areas [96], including vascular tissue [114] and in vitro pharmacology [115], as well as tumor models [116]. Extrusion 3D bioprinting can manufacture complex geometric shapes of vascular disease symptoms, such as bifurcation and curvature [117]. Jakab et al. utilized EBB biotechnology to successfully print tissue globules consisting of human vascular endothelial cells (HUVECs) and heart cells isolated from chicken embryonic cardiac tubes [118]. Jang et al. used EBB to investigate stem cell-laden decellularized extracellular matrix bioinks for fabricating pre-vascularized and functional multi-material structures. They used heart tissue-derived dECM as a bioink to enhance delivered cells [119]. The printed structure, consisting of a spatial pattern of dual stem cells, improved cell interaction and differentiation and promoted tissue regeneration. In order to achieve the preparation of functional vascular models in vitro, the collaborative work of multiple nozzle extrusion can print complex structures. Pati et al. used a complex multi-nozzle 3D bioprinter to distribute different cell-laden decellularized ECM mixtures in isotropic polymer scaffolds [120]. They focused on the development and characterization of bioinks (decellularized ECMs), as well as biological studies of cell viability, apoptosis, and differentiation. In the same field of study, based on a multilayered coaxial nozzle printer, Jia and scholars achieved the direct fabrication of highly organized, perfusable vascular structures using a blend bioink that was designed as an especially cell-responsive bioink consisting of GelMA, sodium alginate, and 4-arm poly (ethylene glycol)-tetra-acrylate (PEGTA) [121], and they improved the coaxial nozzle 3D printer using different sizes of needles. The one-step 3D bioprinting strategy addressed some problems such as creating highly organized 3D vascular networks. In addition, EBB technology effectively solves the manufacturing of organotypic cancer tissue models [122,123]. EBB that precisely locates tissue structures may address some of the challenges of current 3D tumor models in basic biological research and drug screening [124].

EBB technology has led to the development of complex organotypic in vitro models, driving its use in a variety of biomedical applications, such as tissue modeling [125,126] and disease pathophysiology [127,128]. EBB has great advantages in constructing and precisely locating multiple cell types, enabling the manufacture of indigenous tissues in a heterocellular microenvironment. One of the main advantages of EBB is its ability to deposit very high cell densities. However, there are some limitations for EBB. For example, excessive mechanical pressure on encapsulated cells results in reducing cell viability, especially in printing highly viscous bioinks [127], and lower printing speeds are also detrimental to cell survival. Especially, an important but limited part of using EBB to fabricate organotypic constructs is the choice of materials or bioinks [89]. For instance, synthetic materials such as ECMs lack biometric domains, resulting in limited cell–matrix interactions. There is also a huge challenge in terms of bioprinting precision. Although printing precision can be improved by optimizing nozzle/needle size, more attention should be paid to the effects of shear stress on cell viability during printing. Therefore, printing precision, printing speed, and the extracellular matrix should be considered to improve biological 3D bioprinting technology.

#### 4.3.4. Photopolymerization-Based Bioprinting

Photopolymerization-based bioprinting (PBB) is a technical method for fabricating three-dimensional patterned scaffolds with micro/nanostructures. It is a typical light-assisted direct printing method for curing photosensitive biologic inks in the layer-by-layer process. It is a projection printing system that uses a light projector to cross-link photocurable bioinks plane-by-plane [129,130]. Like the components of the LAB system, PBB also requires a light source to solidify the biomaterials. Depending on the light source used to cure the polymer, PBB can be divided into stereolithography (SLA), curing the polymer by laser; digital light processing (DLP), curing the polymer by projector; and continuous digital light processing (CDLP)/continuous liquid light processing (CLIP), curing the polymer by oxygen and light-emitting diodes (LEDs) [131,132]. Moreover, PBB is typically equipped with a set of UV lights, a movement table or bioprinting probe, a material tank, and a computer [133,134]. PBB technology based on ultraviolet (UV) light rapid curing technology can achieve complex geometry and small-size model manufacturing, which makes up for the shortage of extrusion 3D bioprinting technology to a certain extent [135,136]. In the field of vascularization, the most common photoinitiators are Irgacure 2959 (maximum effective wavelength 275 nm) and phenyl-2,4, 6-trimethylbenzoyl phosphate (LAP, maximum effective wavelength 375 nm), which are less cytotoxic [132]. The printing accuracy of PBB technology is high, and the thickness of one layer of photocured material can reach several microns. The high resolution of the micron-scale (≤100 μm) and the very high cell viability (>90%) are also two main characteristics of PBB. With the development of technology, PBB also can print multiple materials simultaneously and locally cure materials.

In the wide applications of PBB technology in biological manufacturing, as one of the important bio-materials in biological tissue engineering, hydrogel is often used in photocurable 3D printing to manufacture vascular tissue. Ma et al. developed a new triculture model based on 3D hydrogels by using PBB technology, which combined physiologically relevant cells with liver-specific 3D microstructures [134]. They embedded hiPSC-derived hepatic progenitor cells (hiPSC-HPCs) as well as endothelial and adipose-derived stem cells into three-dimensional hexagonal hydrogel structures for printing a liver tissue in vitro model, which could improve the human-induced pluripotent stem cell structure and function of hepatic progenitor cells, and thus could be used for early individualized drug screening and the study of in vitro liver pathophysiology. Ye et al. used GelMA hydrogel as a bioink to print and manufacture a multi-channel neural catheter stent based on PBB technology, which provided a new idea and research method for the rapid and accurate printing of NGCs with a complex bionic structure [137]. Grigoryan et al. used stereolithography to build prescribed biomimetic and multivascular architectures using soft hydrogels [138]. They initially studied and analyzed synthetic and natural food dyes as potent biocompatible photoabsorbers to enable stereolithographic hydrogels. Aqueous pre-hydrogel solutions containing tartrazine, curcumin, or anthocyanin could each yield hydrogels with a patent vessel in their report. After determining the hydrogel contents, Grigoryan et al. successfully printed complicated vascular networks based on mathematical space-filling and fractal topology algorithms. These topological vascular models in vitro had complexly entangled networks and could mimic the precise distribution and morphology of blood vessels in the human body.

Due to the ability to prepare complex structures with a high, precise resolution, its applicability to a variety of bioinks, and its computer-aided design and imaging capabilities to generate patient-specific complex tissue [133], PBB technology opens up promising paths in regenerative medicine and disease modeling. An advantage of photopolymerization is the possibility of using low-viscosity resins, which can improve resolution [78]. However, in the PBB method, liquid prepolymers must be added to build the next layer, which takes a relatively long time compared to the exposure time and can generate shear forces on the cells. In addition, cell damage is caused by oxidative stress caused by laser and photoinitiator activation. Cells may become inactivated due to relatively long exposure to UV light during the process [111]. Therefore, in the future development of PBB technology, improving the resolution and shortening the unnecessary exposure time should be considered.

Overall, 3D bioprinting opens up promising avenues in regenerative medicine and disease modeling by promoting the versatility of materials and precise spatial resolution and may provide the necessary technologies for personalized medicine applications when combined with patient-derived cells, custom bio-inks, and microfluidics/sensing technology [139]. As more bioprinting technologies are researched, printing resolution and quality will eventually improve, providing the ability to print more complex 3D structures.

## 5. Advanced Arterial Disease In Vitro Models

Diseased arteries, including atherosclerosis, thrombosis, aneurysm, etc., are caused by changes in the structure and function of blood vessels. Despite clinical advances in the treatment of vascular disease, the mechanisms underlying the progression and regression of multifactorial arterial wall disease are still not fully understood [140,141]. The establishment of vascular disease models in vitro is conducive to understanding and studying its pathology, and provides a relatively convenient biological environment for the treatment of vascular diseases [2].

### 5.1. Atherosclerosis

**Figure 6:**
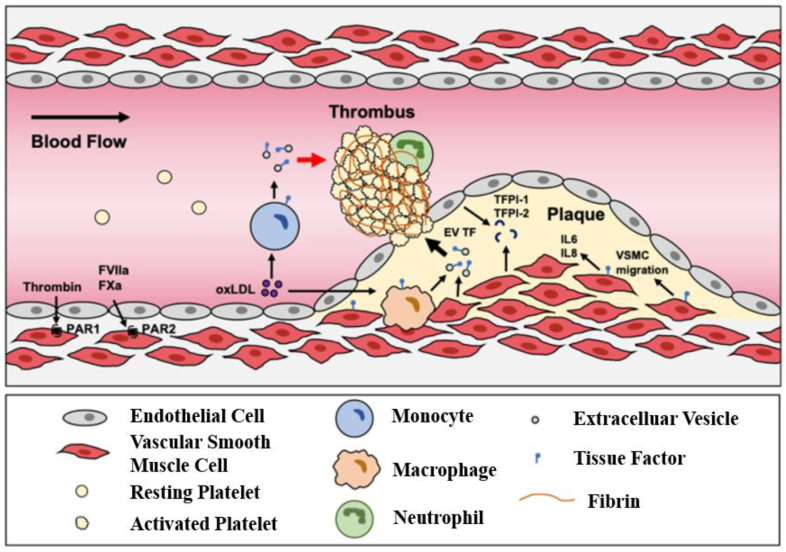
Initiation and progression of atherosclerosis. Reprinted with permission from ref. [142]. Copyright 2022 Copyright Grover et al.

Atherosclerosis begins with endothelial injury induced by inflammatory factors, with large amounts of cholesterol deposited in the major and medium arteries (in Figure 6 [142]). This accumulation leads to the proliferation of cells such as macrophages and smooth muscle cells (SMCs) [143,144]. Most schemata of atherosclerosis initiation suggest that oxidized LDL particles are ligands for clearance receptors that promote foam cell formation. Oxidized LDL particles may induce inflammation and provide new epitopes that stimulate humoral and adaptive immunity [145]. In particular, the endothelial integrity of blood vessels is important for blood circulation. Once endothelial integrity is destroyed, some cells and lipids gather in the subendothelial space. Monocytes differentiate into macrophages, which engulf lipids and differentiate into foam cell macrophages. When foam cells accumulate a large amount of lipids, a necrotic core forms, forming a fibrin cap that becomes an atherosclerotic plaque. Within the artery wall, the expansion of atherosclerotic plaque can gradually attack the blood vessels and block blood flow [146]. Once the plaque ruptures, it will release some lipids and contents as acute thrombosis, causing embolism in specific areas, particularly blocking vital blood vessels of the heart and brain, which leads to ischemic heart disease and stroke.

Undoubtedly, the initiation and progression of atherosclerosis is a complex process entailing a variety of cellular responses and interactions, as well as dynamic biomechanical and biochemical stimulations. Upon the development of biofabrication techniques, diverse, advanced in vitro models have emerged in recent years, which can recapitulate the pathophysiology of atherosclerosis at a higher level in comparison with conventional platforms. Zheng et al. used a microfluidic platform to simultaneously apply fluid shear stress and cyclic stretching to endothelial cells to reproduce the mechanical conditions associated with early atherosclerosis [147]. Endothelial cells cultured in the facility were treated under physiological flow and circulatory stress conditions with Probucol, a drug withdrawn from the market due to off-target cardiovascular effects, and cardiotoxicity not seen in conventional cell culture models was found. They further showed that their device produced similar results in mouse models after treating hyperglycemia with antioxidant-functionalized nanoparticles.

Designing a 3D acellular scaffold followed by the seeding of different cells to fabricate tissue-engineered arterial models for the investigation of arterial disease in vitro is another remarkable idea. Truskey et al. designed a tissue-engineered blood vessel (TEBV) model with a three-layer structure, ECs, SMCs, and fibroblasts [148]. Early atherosclerosis closely connects with endothelial dysfunction; low-density lipoprotein (LDL), tumor necrosis factor-alpha (TNF-α), and monocytes are some risk factors for facilitating this form of atherosclerosis. They perfused a model with these, mimicking the early development of endothelial injury. Increased concentrations of enzyme-modified LDL lead to foam cell formation, monocyte migration, and accumulation, which form vessel inflammation. Robert et al. reported a dynamic artery model in vitro. The perfused nutrient medium combined some risk factors such as low-density lipoprotein (LDL), monocytes, and tumor necrosis factor-alpha (TNF-α), and acquired LDL accumulated in the sub-endothelial space, which is an early development of atherosclerosis [149]. They used polyglycolic acid to fabricate a 3D tubular scaffold, seeded human umbilical cord-derived myofibroblasts (UCMFBs) in the lumen inner surface, and utilized fibrin as the cellular carrier, cultivated for 3 days. The construct stayed in the flow bioreactor system for 14 days, then human umbilical vein ECs (HUVECs) were seeded in the inner surface and cultured in a static environment for 5 days. Finally, this vascular graft was incubated in a flow bioreactor system for further research. These processes offered a dynamic flow simulation but did not consider the surrounding environment; all cells and arteries live in an internal environment and communicate with the external matrix.

Compared with the scaffold-based approach, 3D bioprinting can pave the way to easily achieving the goal of engineering arterial analogs in vitro. Notably, since the artery is a multi-layer lumen structure, utilizing coaxial bioprinting could effectively establish the model. Gao et al. reported an advanced bioprinting strategy for a triple-layer artery model with lumen-regulating diameters, geometry-tunable by controlling the nozzle moving speed, flow rate, and pneumatic pressure [74]. The triple coaxial nozzle used calcium Pluronic F127 (as sacrificial materials) in the core nozzle, vascular tissue-derived decellularized extracellular matrix (VdECM)-encapsulated HUVECs in the middle nozzle, and VdECM-encapsulated human coronary artery smooth muscle cells (HCASMCs) in the shell nozzle. The construct was bioprinted in an optimized bath ink (as surrounding tissue matrix and supporting materials) that was composed of 3% VdECM and human dermal fibroblasts (HDFs). The perfused turbulent flow artery model with a high concentration of LDL fabricated an early atherosclerosis model. They also used the model to evaluate the effects of Atorvastatin, a drug that lowered cholesterol. Obviously, the 3D bioprinting atherosclerosis model as an assistant tool has extensive application for studying the development of the disease and exploring the effectiveness of drugs. They fabricated the multi-layer artery structure by an interesting method and made the structure live in a 3D matrix, which mimicked the artery native environment.

In addition, an artery/atherosclerosis model on a chip has been established for studying the development of atherosclerosis [150]. Su et al. first reported a microfluidic EC-SMC co-culture device for a normal artery wall model. Then they stimulated the model with pro-inflammatory cytokines (such as TNF-α and IL1-β) and oxidized LDL for studying endothelial inflammation in early atherosclerosis [151]. Furthermore, drug screening was also applied in this model and confirmed that Vitamin D, a drug known as anti-atherogenic, could inhibit monocytes–EC adhesion and SMC migration. From another review, EC-SMC co-culture devices had developed a lot and could be used for high-throughput drug screening [148].

All in all, regardless of which biotechnology is used to construct in vitro models of atherosclerosis, there are key points that need to be considered to improve the accuracy of in vitro models and gain insight into this systemic disease. First and foremost, cells carrying atheroprone mutations should be considered in the atherosclerosis of in vitro models to better understand each mutation’s peripheral impact on the vasculature. Second, it is important to fabricate an in vitro model of atherosclerosis tailored to the patient for diagnosis, which is conducive to achieving target-oriented treatment. In particular, atherosclerotic models should predict plaque size, location, and growth rate based on patient data to predict disease prognosis and provide a successful impact on specific treatments [2].

### 5.2. Thrombosis

**Figure 7:**
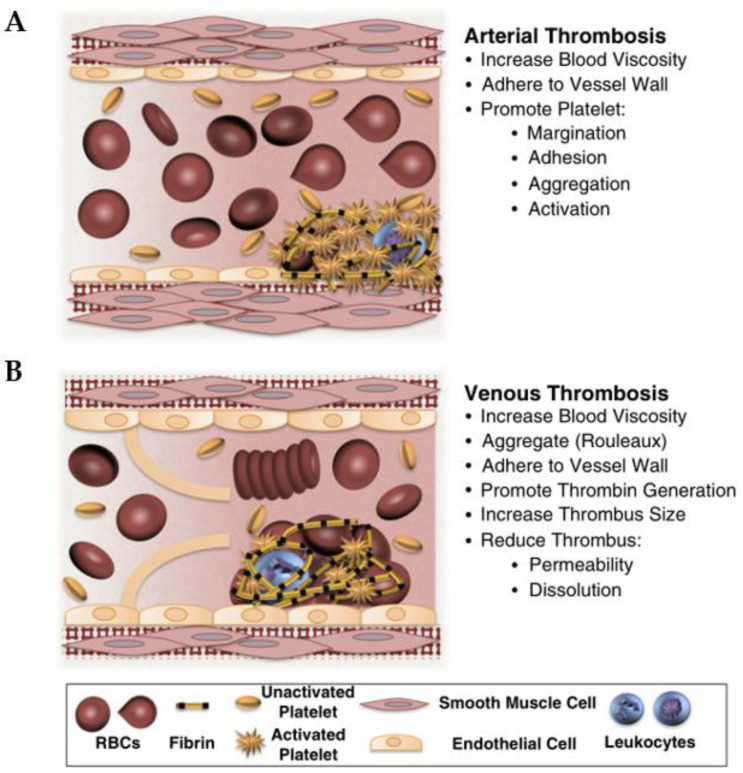
Pathological thrombosis: (**A**) arterial thrombosis and (**B**) venous thrombosis. Reprinted with permission from ref. [153]. Copyright 2022 Copyright Byrnes et al.

In a healthy body, clotting and anticoagulation systems function normally. When vessel wall injury, platelets, tissue factor, and fibrin accumulate, they form a thrombus [152]. Atherosclerosis plaques rupture and release the contents as arterial thrombosis, leading to the blockage of the small artery, as referred to earlier. Vein thrombosis is usually caused by slow blood flow, damage to blood vessel walls, and hypercoagulable states, which include two types. One occurs in patients with prolonged inactivity of the lower extremities, called venous thrombosis. The other is arterial thrombosis, which is caused by the rupture of atherosclerotic plaque, causing the complete or partial occlusion of blood vessels. When a plaque ruptures, its lipid core is exposed to circulating blood in the artery’s lumen (in Figure 7) [153,154].

Thrombosis-on-a-chip is a useful model for studying developing blood clots due to utilizing human ECs and human blood for studying interactions, which is rare in other technology [155]. Zhang et al. reported utilizing 3D bioprinting to generate a thrombosis-on-a-chip, in which PF127, used as a sacrificial material for form microchannels, and GelMA, used as a matrix, fabricated a bifurcate channel model [156]. HUVEC was seeded into microchannels to form confluent endothelium, then perfused human whole blood was perfused with 10 vol% 0.1 M CaCl_2_ solution, added to induce clotting. Ten minutes later, they observed the clotting formation and H&E staining confirmed thrombus formation. They perfused this model with a tissue plasminogen activator, a thrombolytic agent, and 7 days later achieved a good thrombolytic effect. In addition, they explored the fibroblast existence for the formation of fibrotic clots.

Microfluidic devices can mimic physiological fluid shear and achieve changeable geometry, which is important for clinical experiments [83]. Jain et al. reported chemically fixed ECs to explore the thrombosis form and platelet adhesion. The model offered a physiological shear and had the potential for in vitro diagnosis [157]. Zheng et al. used collagen I to fabricate a microfluidic vessel network and seeded ECs on the surface [158]. After long-term culturing, the endothelial function of the vascular network was well established. The perfusion of human whole blood was used to mimic the flow of normal blood vessels. Integrity endothelium offered an appropriate microenvironment for blood platelets and platelets incapable of adhesion to the vessel wall. This model was stimulated by brief exposure to phorbol-12-myristate-13-acetate (PMA), inducing platelet adhesion to the surface of the vessel model and platelets aggregating into clumps. Costa et al. utilized computed tomography angiography (CTA) data combined with stereolithography (SLA) 3D bioprinting technology to fabricate a normal 3D vessel structure model and a stenotic model [159]. HUVEC seeded in the surface of the channel mimicked the flow of the normal blood. They perfused the channel with human citrated whole blood; the normal vessel model exhibited normal blood flow and the stenotic vessel model exhibited platelet aggregation and thrombosis formed after 2 min.

In conclusion, all in vitro models of diseases are designed to accurately express pathological processes to provide targeted treatment options. Thrombosis, a common and fatal blood vessel disease, should be considered precise and reproducible in 3D in vitro models. Key cell populations, ECM components, morphology, and the intravascular topography involved in coagulation cascade and hydrodynamics should be considered in the preparation of its in vitro model to better express the coagulation pathological process of thrombus.

### 5.3. Aneurysm

**Figure 8:**
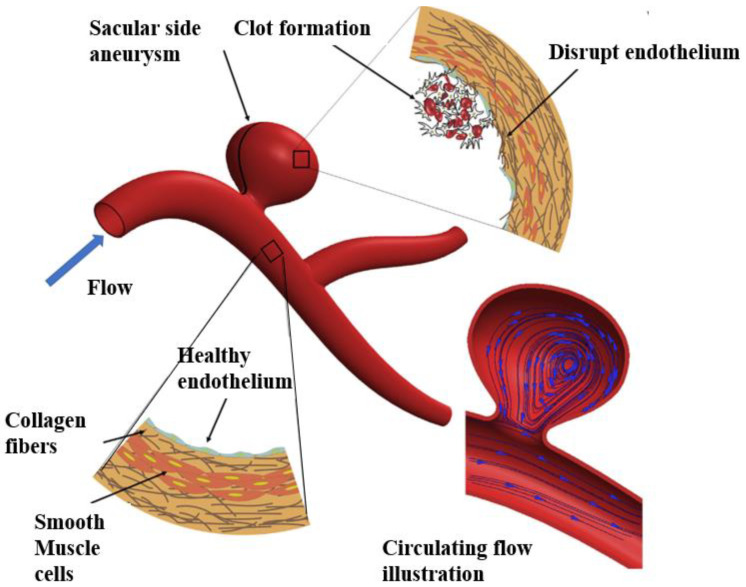
Association of aneurysms with abnormal hemodynamics. Reprinted with permission from ref. [168]. Copyright 2022 Copyright Byrnes et al.

The aneurysm is defined as a local globular dilation of a normal artery that is about 1.5 times larger than the diameter of a normal adjacent segmentary artery [160,161]. Clinically, atypical aneurysms can be divided into three types [106]: (1) the expansion of the entire tube wall and lumen, namely mycotic aneurysm; (2) a laminar tear in the wall of the tube that causes the lumen to widen, called a dissecting aneurysm; (3) the blood vessel ruptures, the sub-adventitia hematoma forms a tumor-like dilation, and the tumor expands outside the wall, though the true lumen is not large, which is a pseudoaneurysm [162]. Aneurysms occur in cerebral arteries [163], pulmonary arteries [164], and abdominal arteries [165], and are fatal diseases caused by the deformation and remodeling of blood vessel walls. Aneurysms are seriously affected by hemodynamics [166,167]. Pathological hemodynamics are prevalent in aneurysms, such as low shear recirculation flow that activates and destroys endothelial cells. In addition, pathological hemodynamics in aneurysms lead to mass transport and granular dynamics, which are significantly different from features in normal vessels and contribute to disease progression. Besides, at the local site of a ruptured aneurysm, the interaction between damaged or activated endothelial cells and flowing blood cells (platelets or white blood cells) is closely related [168]. Figure 8 shows aneurysms associated with the abnormal hemodynamics of pathobiological processes. Due to the lack of suitable in vitro aneurysm models to simulate the pathophysiological conditions causing the disease, detailed studies have not been carried out. Therefore, when using biotechnology to prepare aneurysm models in vitro, it is necessary to expand and explore new treatment methods for aneurysms based on the principle of the aneurysm formation mechanism [1,169].

The microfluidic platform can intuitively simulate the pathological hemodynamics of aneurysm in vitro models and mimic the influence of aneurysm morphology on aneurysm formation [170]. Kaneko et al. reconstructed aneurysms using a 3D microfluidic model of hydrogel and used patient MRI scans to inform 3D printing, generating the structure of the device during manufacturing [171]. Their microfluidic device confirmed low wall shear stress in the aneurysm sac. Additionally, when exposed to fluid flow, endothelial cells in aneurysms were shaped differently from endothelial cells in the lumen. Similarly, Mannino et al. also used a three-dimensional microfluidic model and hydrogel to reproduce aneurysms based on a two-dimensional flow model and confirmed that the shear stress in the aneurysm sac was low [172]. To enable the flow signals in a microfluidic platform, peristaltic or syringe pumps are usually necessary accessories. However, the pump-driven circulating system inevitably causes complexity of the system. To overcome this challenge, a pumpless microfluidic device that relies on gravity-induced flow has been developed and applied for the establishment of vascular disease models. Hosseini et al. designed a simple, pumpless, closed-loop, easily replicable, and miniaturized flow device that could simultaneously expose 3D engineered vascular smooth muscle tissue to study the aneurysm correlation of changing blood flow patterns under high speed pulsating flow and low-speed disturbance flow conditions [173]. They found that laminar high-speed flow can promote the development of healthy ECMs. On the contrary, low velocity disturbed flow promotes ECM aggregation. Although the microfluidic platform has achieved great research in simulating aneurysm models in vitro, this method still has limitations. The microfluidic platform is frequently used to study the effect of hemodynamics on the aneurysm and to simulate the pathological reaction of aneurysm cells under the condition of real pulsating blood by adding mechanical equipment externally. There is still a need to improve microfluidic platforms in pathophysiological conditions, drug targeted therapy, aneurysm evaluation, and other aspects.

Compared with other methods of in vitro model manufacturing, 3D bioprinting is a more promising method. Three-dimensional bioprinting technology can build a more accurate 3D aneurysm model to better reproduce the direct effect of blood flow on the aneurysm. In this way, the in vitro model of the aneurysm can be established, which is beneficial to further understanding the pathology of aneurysms and predicting the deformation and strain degree of aneurysm vessels. Importantly, 3D bioprinting technology can realize the research of drug targeted therapy for aneurysms. Epshtein et al. established an in vitro model of aneurysms by using photocurable 3D bioprinting technology [168]. They developed a particle carrier designed to locate the low-shear flow of aneurysms and adhere to the vascular wall of lesions. The experimental results demonstrated that glycoprotein VI could effectively target the aneurysm site. Their proposed biophysical strategy of targeted delivery may provide new therapeutic opportunities for brain aneurysms. In recent years, patient-specific treatment for patients has become a research hotspot, and 3D bioprinting has the potential to provide technology for patient-specific treatment. Modero et al. used 3D bioprinting to fabricate a patient-specific aneurysm in vitro model based on a computed tomography (CT) angiogram [174]. They chose water-dissolvable material as the printed material and submerged the model in silicone. They assessed the circulatory hemodynamic changes in a patient-specific intracranial aneurysm model using a tomographic particle image velocimeter at high imaging rates under pulsating flow conditions. Especially, as 3D bioprinting technology can prepare models of aneurysm lesions of different degrees, it provides a medical diagnostic technology for predicting aneurysms at different stages. Jang and teammates proposed the first-ever in vitro living 3D printed aneurysm with a fully encapsulated extracellular matrix interrogation therapy and intravascular device [175]. This in vitro aneurysm model could test the biocompatibility and hemostatic efficiency of the embolization device and provide hemodynamic information for predicting aneurysm rupture or healing response after treatment. Furthermore, Gao et al. assembled an in vitro model of an artificial vascular framework based on 3D bioprinting for the early diagnosis of intracranial aneurysms [176]. They first made transparent artificial blood vessel models at six different growth stages using 3D bioprinted hyper assembly technology. Then, phase-contrast magnetic resonance imaging (PC-MRI) and computational fluid dynamics (CFD) techniques were used to evaluate the flow field during IA growth. This method has the potential for the early diagnosis of aneurysms.

Summarily, more accurate aneurysm in vitro models should be developed to better reproduce the direct effect of blood flow on the formation of new ECMs. Combining 3D bioprinting and microfluidic technology, further study on the relationship between fluid dynamics and the stress–strain of aneurysm vessels, as well as the remodeling of cells such as ECs at the location of lesion vessels under the action of blood fluid. Importantly, an appropriate in vitro model of aneurysms should also facilitate discussion of what cellular, extracellular, and mechanical signals promote the synthesis and regulation of proteins involved in ECM remodeling.

## 6. Conclusions

The development of diseased artery in vitro models from 2D static to 3D dynamic is an important embodiment of the cross-era of biotechnology, which is conducive to a better understanding of pathology and the realization of drug therapy. As the basis for the construction of arterial disease in vitro models, it is important to understand the physiological elements in the target tissue, including cells, ECMs, and dynamic signals, as well as the interactions between them. Although a variety of conventional techniques, such as designed apparatuses, transwell systems, and needle-templating microchannels have been developed to model the arterial pathology in vitro, their limited ability to precisely mimic the natural cellular, biomechanical, and biochemical cues confined their applications. To overcome these challenges, advanced biofabrication technologies, microfluidic platforms, organ-on-a-chip, and 3D bioprinted artery analogs emerged and made a qualitative leap in the in vitro models of artery-relevant diseases, including atherosclerosis, thrombosis, and aneurysm. The accumulated achievements provide a better understanding of the pathology of human arterial disorders and suggest a novel tool for drug discovery.

In the future, the technology for realizing the in vitro modeling of a human artery is still the hot research in the field of biofabrication, especially for the in vitro modeling of patient-specific diseased tissue. This may require a combination of medical imaging, organ-on-a-chip, and 3D bioprinting to construct a sophisticated system that can mimic natural artery tissues.

## Data Availability

Not applicable.
